# Minimizing blood loss in laparoscopic myomectomy with temporary occlusion of the hypogastric artery

**DOI:** 10.3389/fmed.2023.1216455

**Published:** 2023-08-22

**Authors:** Ligia Balulescu, Samuel Nistor, Diana Lungeanu, Simona Brasoveanu, Marilena Pirtea, Cristina Secosan, Dorin Grigoras, Radu Caprariu, Andrea Pasquini, Laurentiu Pirtea

**Affiliations:** ^1^Department of Obstetrics and Gynecology, Victor Babes University of Medicine and Pharmacy, Timisoara, Romania; ^2^Department of Obstetrics and Gynecology, Timisoara Municipal Emergency Clinical Hospital, Timisoara, Romania; ^3^Center for Modeling Biological Systems and Data Analysis, Victor Babes University of Medicine and Pharmacy, Timisoara, Romania; ^4^Institute of Cardiovascular Diseases, Timisoara, Romania; ^5^Department of Functional Sciences, Victor Babes University of Medicine and Pharmacy, Timisoara, Romania; ^6^Department of Radiology and Medical Imaging, Victor Babes University of Medicine and Pharmacy, Timisoara, Romania; ^7^“Pius Brinzeu” County Clinical Emergency Hospital, Timisoara, Romania

**Keywords:** blood loss, temporary occlusion of the hypogastric artery (TOHA), clipping, operative time, laparoscopic myomectomy, uterine leiomyoma, uterine myomectomy

## Abstract

**Introduction:**

Uterine leiomyomas are common benign pelvic tumors. Currently, laparoscopic myomectomy (LM) is the preferred treatment option for women in the fertile age group with symptomatic myomas. The authors hypothesize that combining LM with a bilateral temporary occlusion of the hypogastric artery (TOHA) using vascular clips minimizes uterine blood flow during surgery and can significantly reduce surgery-associated blood loss.

**Materials and methods:**

This single-center, prospective randomized study was conducted at the Department of Obstetrics and Gynecology, Municipal Emergency Clinical Hospital Timisoara, Romania. Patients aged between 18 and 49 who preferred laparoscopic myomectomy and wished to preserve fertility were included, provided they had intramural uterine leiomyomas larger than 4 cm in diameter that deformed the uterine cavity. The study analyzed data from 60 laparoscopic myomectomies performed by a single surgeon between January 2018 and December 2020. Patients were randomly assigned to either: “LM + TOHA” group (29 patients), and “LM” group (31 patients). The study’s main objective was to evaluate the impact of TOHA on perioperative blood loss, expressed as mean differences in Hb (delta Hb).

**Results:**

Delta Hb was statistically lower in the “LM + TOHA” group compared to “LM” group, with mean ± standard (min–max): 1.68 ± 0.67 (0.39–3.99) vs. 2.63 ± 1.06 (0.83–4.92) g/dL, respectively (*p* < 0.001). There was a statistically significant higher need for postoperative iron perfusion in the “LM” group, specifically 0 vs. 12 patients (*p* < 0.001), and lower postoperative anemia in “LM + TOHA” group (*p* < 0.001). Necessary artery clipping time was 10.62 ± 2.47 (7–15) minutes, with no significant impact on overall operative time: 110.2 ± 13.65 vs. 106.3 ± 16.48 (*p* = 0.21). There was no difference in the length of hospitalization or 12-month post-intervention fertility.

**Discussion:**

Performing bilateral TOHA prior to laparoscopic myomectomy has proven to be a valuable technique in reducing surgery-associated blood loss, while minimizing complications during surgery, with no significant increase in the overall operative time.

**Clinical trial registration:**

ISRCTN registry, (www.isrctn.com), identifier ISRCTN66897343.

## 1. Introduction

Uterine leiomyomas, also referred to as fibroids or myomas in the literature, are the most frequently occurring solid benign pelvic tumors in women (1–3). It has been estimated that among Caucasian women in the United States, lifetime incidence of uterine leiomyomas is as high as 40% by the age of 35, and exceeds 70% among women aged 50 and above (4). Reported prevalence of diagnosed uterine fibroids among five European countries ranged between 11.7% in France to 23.6% in Italy (4).

Leiomyomas, frequently asymptomatic and often diagnosed incidentally, can result in complications given by the compression or displacement of adjacent pelvic organs such as pelvic pressure, pain, increased urinary frequency, constipation, as well as local implications, such as irregular or excessive menstrual bleeding, recurrent pregnancy loss, and even infertility (5).

Laparoscopic myomectomy (LM) is a minimally invasive and fertility-preserving procedure which has several advantages over laparotomy, such as reduced postoperative pain, a shortened hospital stay and recovery time, and a decreased risk of adhesion formation (6, 7). Therefore, it is the current preferred method for performing a myomectomy (8–10).

Surgery that involves the uterus and myomas can lead to substantial blood loss because of their rich vascularity, irrespective of surgical approach (11). To address this concern and minimize intraoperative bleeding, several techniques have been developed over time. These can be either: (a) non-surgical, such as intramyometrial injections with vasopressin, or the use of misoprostol or tranexamic acid; or (b) surgical, including the use of a pericervical tourniquet, permanent uterine artery occlusion via bipolar coagulation, uterine artery embolization, and a recent combination approach that involves LM and a temporary uterine artery occlusion (TUAO) with sutures or clips (12–14). This article introduces a new technique of LM combined with temporary occlusion of the hypogastric artery (TOHA). This technique involves the bilateral clipping of the anterior trunk of the hypogastric artery (also known as the internal iliac artery) cranially to uterine artery emergence. Authors underline the paucity in the literature of this specific approach for temporary clipping during LM.

This study aims to investigate the effectiveness of TOHA in reducing surgery-associated blood loss during laparoscopic myomectomy.

## 2. Materials and methods

### 2.1. Study design and data collection

This research article was designed as a single-center, prospective randomized study, following the Consolidated Standards of Reporting Trials (CONSORT) guidelines (15, 16).

To evaluate the impact of performing a concomitant temporary occlusion of the hypogastric artery during LM, 62 patients were randomly allocated to one of two groups: 31 patients who underwent LM and TOHA with clipping (“LM + TOHA” group), and 31 patients who benefited from standard LM without clipping (“LM” group). After randomization, two patients from the LM + TOHA group did not undergo surgery and were eliminated from the study: one patient had decompensated heart failure diagnosed before surgery, and another had deep infiltrating endometriosis with limited access to the left side wall. Follow-ups were scheduled at two weeks, six weeks, six months, and one year after surgery.

This study was reviewed and approved by the Human Ethical Committee of the University of Medicine and Pharmacy “Victor Babes,” Timişoara, Romania (Nr 44/10.12.2018). It was conducted between the 1st of January 2018 and the 31st of December 2020 at the Municipal Emergency Clinical Hospital, Timisoara, Romania.

### 2.2. Patient selection and characteristics

The main indication for myomectomy was abnormal uterine bleeding. Inclusion criteria were: (a) patients aged between 18 and 49; (b) patient preference for laparoscopic myomectomy and their desire to preserve fertility; and (c) patients who had intramural uterine leiomyomas greater than 4 cm in diameter, which also deformed the uterine cavity.

**Figure 1:**
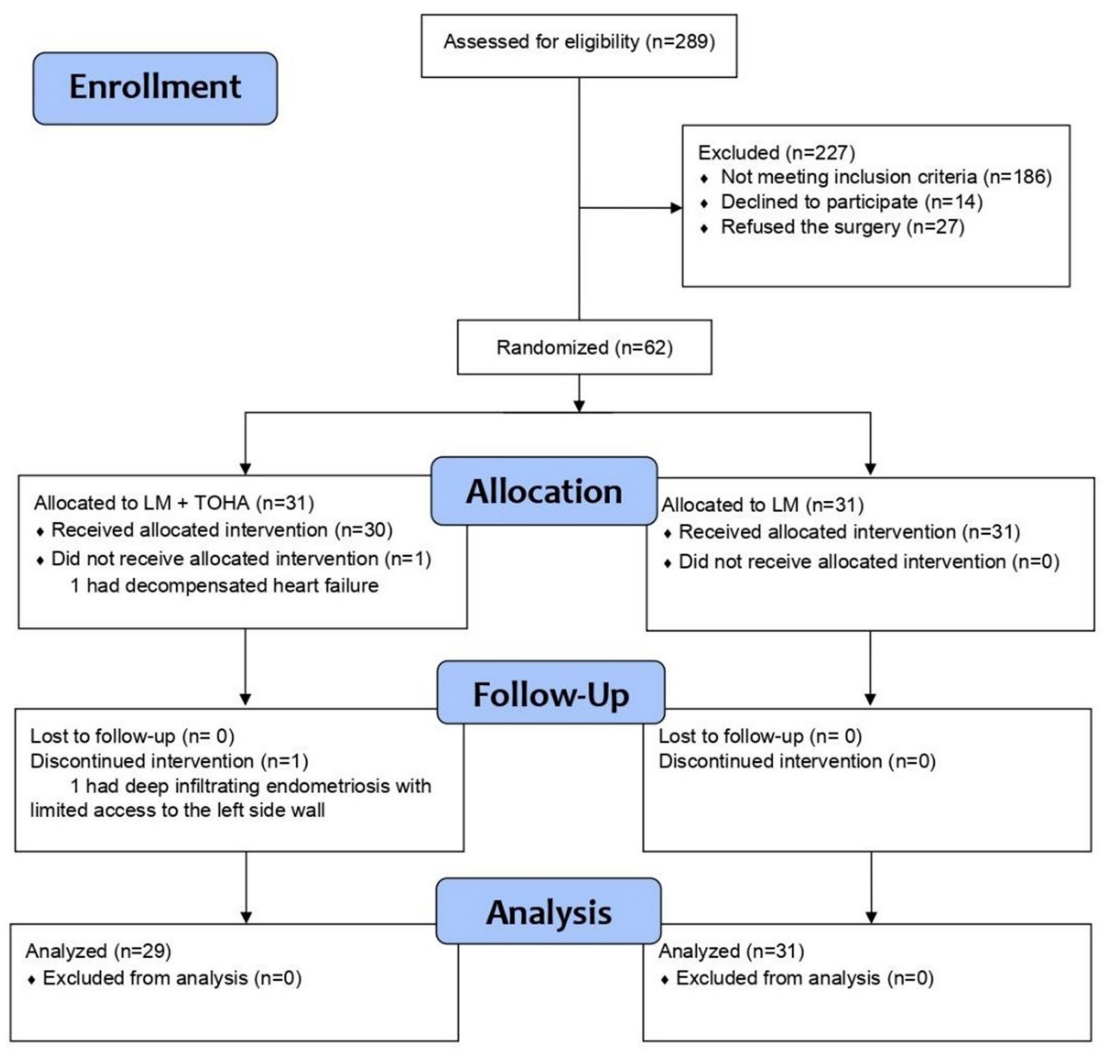
Diagram of patients who underwent laparoscopic myomectomy, according to CONSORT 2010.

Exclusion criteria were as follows: (a) patients who did not agree to the enrollment or did not pass inclusion criteria (such as: age over 50, no preference for fertility preservation, personal option for hysterectomy); (b) cases with other types of myomas (such as submucosal or subserosal location), or intramural myomas under 4 cm which did not have an impact on the uterine cavity; and (c) cases suspected of malignancy. Aforementioned criteria were represented in the CONSORT flowchart (Figure 1).

Necessary sample size was determined based on the primary objective of comparing blood loss and surgery duration (namely a *t*-test for independent samples). The analysis used a power of 0.9, alpha level of 0.05, two-sided alternative, and Cohen’s d, effect size of 0.9. The calculation indicated a minimum of 27 patients in each group. To account for potential dropouts, a 15% coefficient was applied, resulting in a final required sample size of 31 patients in each group. Randomization was single-blind and performed with the R package “blockrand” v. 1.5. Two TOHA patients were lost after randomization to the treatment groups. As this was a surgical investigation, on-treatment analysis of data was conducted, so the final groups were of unequal sizes, but required statistical power was maintained.

Preoperatively, a diagnostic ultrasound examination was performed in order to assess the most suitable surgical approach.

### 2.3. TOHA and LM procedures

All surgical procedures were performed by a single and highly proficient surgeon with over a decade of experience in laparoscopic surgery. Prior to the surgery, neither Gonadotropin-Releasing Hormone (GnRH) agonists nor any intra-operative hemostatic drugs, such as vasopressin injection, were employed. Before initiating surgical procedures, general anesthesia was induced concomitantly with orotracheal intubation.

**Figure 2:**
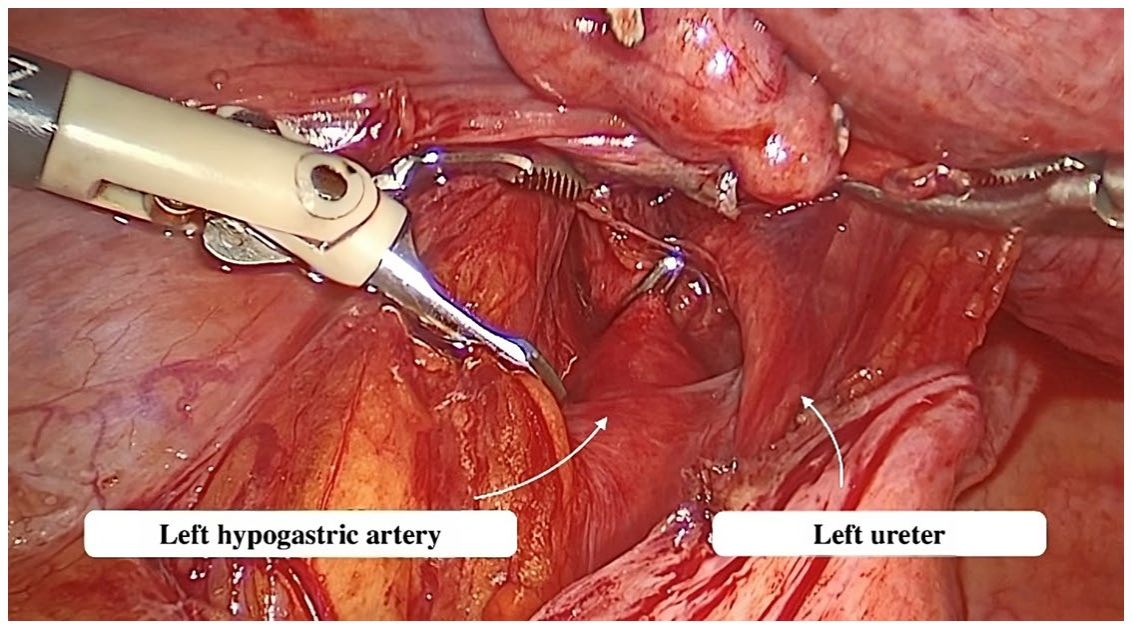
Dissection of the hypogastric artery.

Abdominal access was obtained via a direct trocar entry technique. CO2 insufflation was utilized to create a pneumoperitoneum with a low intra-abdominal pressure of up to 12 mmHg. A single 10 mm trocar was positioned on the median line, 8 cm away from the umbilicus, while two 5 mm trocars were situated on each side of the lower abdomen. The patient was then reclined in a Trendelenburg position. The parietal peritoneum was incised below the lumbo-ovarian ligament. The ureter and anterior trunk of the hypogastric artery were identified in the area where these two structures run parallel (Figure 2). The ureter and hypogastric artery were identified using blunt dissection. A metallic clip was then placed on the anterior trunk of the hypogastric artery, cranially to the uterine artery emergence.

The uterine wall was incised, and the myoma/s were located and then removed using traction and countertraction maneuvers. Next, in-bag morcellation was used to remove the leiomyoma/s. The uterine wall was sutured with a double-layer stitch, utilizing a 29 mm, 3/8 circle needle, and Vicryl 2.0 thread. Once the uterine wall had been closed, hemostatic clips were gently removed by traction using atraumatic Johan fenestrated forceps.

### 2.4. Statistical data analysis

Categorical variables were described by the observed frequencies (i.e., counts) and their corresponding percentages; The Chi-square test was applied for statistical significance (either asymptotic, or Monte-Carlo simulation based on 10,000 samples).

Normality of numerical variables was tested with Kolmogorov–Smirnov statistical test; their descriptive statistics comprised the mean ± standard deviation and (minimum–maximum) interval, irrespective of their distribution. To compare normally distributed series of values, the *t*-test for independent groups was applied for the means, and Levene’s test for the variances. To compare non-normal numerical data across two groups, a non-parametric Mann–Whitney U-test was applied.

The statistical analysis was conducted at a 95% level of confidence (i.e., 5% level of statistical significance). All reported probability values were two-tailed, and statistical significance was explicitly marked. Data were analyzed with the statistical software IBM SPSS v. 20.0 and R v. 4.2.2 (package “pwr”).

## 3. Results

The only clinical differences between the two study groups that proved to be statistically significant were the higher nulliparity rate in the “LM + TOHA” group [18 (62.1%) vs. 9 (29%), *p* = 0.01], and reported symptoms such as infertility, meno-metrorrhagia, and pain (Table 1).

**Table 1:** Clinical data in the two study groups of laparoscopic myomectomy (LM): with and without transient occlusion of hypogastric artery (TOHA).

	LM + TOHA	LM	*p*-value
Variable	*N* = 29	*N* = 31	
Age (years) ^(a)^	34.5 ± 5.2 (26–44)	36.5 ± 5.2 (28–46)	0.136
BMI (kg/m^2^) ^(a)^	26.5 ± 4.2 (17.1–33.2)	27.0 ± 3.7 (18.9–33.2)	0.681
Symptoms			
Infertility ^(b)^	18 (62.1%)	8 (25.8%)	0.005**
Menstrual disorder / Meno-metrorrhagia ^(b)^	13 (44.8%)	22 (71.0%)	0.04*
Pain ^(b)^	9 (31.0%)	21 (67.7%)	0.04*
Nulliparous ^(b)^	18 (62.1%)	9 (29%)	0.01*
Number of leiomyomas ^(c)^	1.31 ± 0.66 (1–3)	1.32 ± 1.14 (1–7)	0.445
Number of leiomyomas/patient ^(d)^			0.483
1	23 (79.3%)	27 (87.1%)	
2	3 (10.3%)	2 (6.5%)	
3	3 (10.3%)	1 (3.2%)	
4	0	0	
5	0	0	
6	0	0	
7	0	1 (3.2%)	
Size of the largest leiomyoma (cm) ^(c)^	7.21 ± 1.66 (5–12)	7.42 ± 2.1 (4–12)	0.77
Location of dominant leiomyoma ^(d)^	38 (100%)	41 (100%)	0.972
Anterior wall	13 (34.2%)	11 (26.8%)	
Posterior wall	15 (39.5%)	18 (43.9%)	
Fundical	10 (26.3%)	11 (26.8%)	
Lateral (intraligamentary)	0	1 (2.4%)	

Data presented as mean ± standard deviation (min-max) for numerical variables, or *n* (%) for categorical variables. ^(a)^ *t*-test for independent samples; ^(b)^ asymptotic Chi-square test; ^(c)^ non-parametric Mann–Whitney U-test; ^(d)^ Chi-square, Monte Carlo simulation (10,000 samples). Statistical significance: *p < 0.05; **p < 0.01. LM, laparoscopic myomectomy; TOHA, transient occlusion of the hypogastric artery; BMI, body mass index.

**Figure 3:**
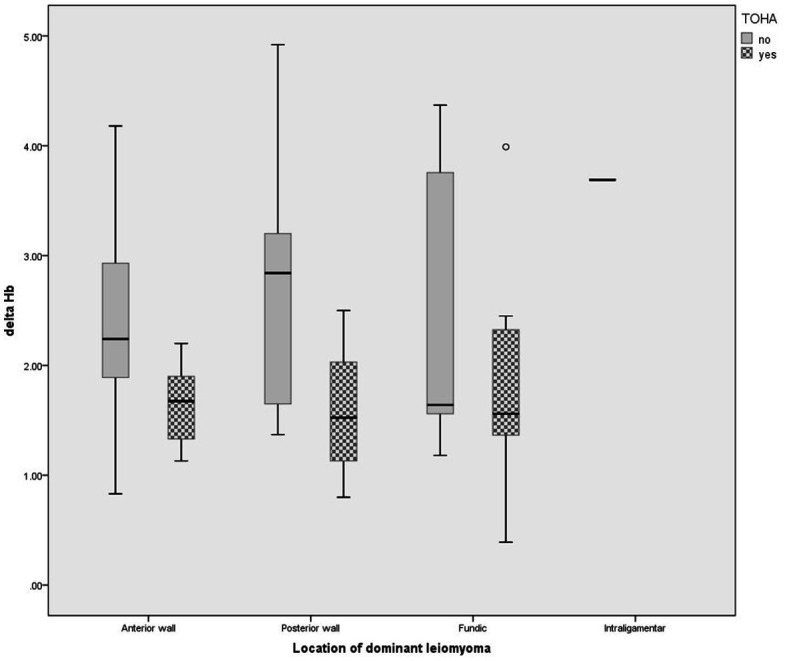
Change in hemoglobin level for different location of dominant leiomyomas in the two study groups of laparoscopic myomectomy (LM): with and without transient occlusion of hypogastric artery (TOHA). The boxes are proportional to the inter-quartile range (IQR) with medians marked between them, and whiskers are proportional to 1.5*IQR (or trimmed to the minimum/maximum values, except for the outlier marked as a bullet).

Statistically significant differences were observed for the levels of preoperative and postoperative hemoglobin (Hb) (*p* = 0.01 and *p* < 0.001, respectively) (Table 2). The significantly lower Delta Hb in the “LM + TOHA” group is apparent for all patients, irrespective of the location of dominant leiomyoma, namely anterior/posterior wall or fundical (Figure 3).

**Table 2:** General surgical data in the two study groups of laparoscopic myomectomy (LM): with and without transient occlusion of hypogastric artery (TOHA).

	LM + TOHA	LM	*p*-value
Variable	*N* = 29	*N* = 31	
History of abdominal surgery ^(d)^	3 (10.3%)	2 (6.5%)	0.256
Conversion to laparotomy ^(e)^	1 (3.4%)	1 (3.2%)	0.962
Pre-operative Hb level (g/dL) ^(a)^	13.23 ± 1.1 (9.5–14.8)	12.23 ± 1.72 (8.78–15.02)	0.01*
Pre-operative anemia ^(d)^	1 (3.4%)	0	0.3
Pre-operative transfusion for severe anemia ^(d)^	1 (3.4%)	0	0.3
Post-operative Hb level (g/dL) ^(a)^	11.56 ± 0.94 (9.11–13.2)	9.60 ± 1.79 (6.01–12.5)	<0.001**
Operative time (min) ^(c)^	110.2 ± 13.65 (90–135)	106.3 ± 16.48 (90–140)	0.21
Clipping length (min)	10.62 ± 2.47 (7–15)	NA	NA
Hospital stay after surgery (days) ^(c)^	2.1 ± 0.6 (1–3)	2.4 ± 0.8 (1–4)	0.076
Myoma removal technique			
Morcellation ^(d)^	28 (96.6%)	25 (80.6%)	0.104
Minilaparotomy ^(d)^	1 (3.4%)	6 (19.4%)	0.104

Data presented as mean ± standard deviation (min-max) for numerical variables, or *n* (%) for categorical variables. ^(a)^ *t*-test for independent samples; ^(b)^ asymptotic Chi-square test;^(c)^ non-parametric Mann–Whitney U-test; ^(d)^ Chi-square, Monte Carlo simulation (10,000 samples); ^(e)^ Fisher’s exact test. Statistical significance: *p < 0.05; **p < 0.01. LM, laparoscopic myomectomy; TOHA, transient occlusion of the hypogastric artery; Hb, hemoglobin; NA, not applicable.

The mean ± standard deviation clipping length of the anterior trunk of the hypogastric artery was 10.62 ± 2.47 min (between 7 and 15 min), with no statistically significant difference in the overall operative time between the two groups: 110.2 ± 13.65 vs. 106.3 ± 16.48 (*p* = 0.21). Moreover, the number of hospitalization days after the procedure was not impacted: 2.1 ± 0.6 vs. 2.4 ± 0.8 (*p* = 0.076) (Table 2).

Histopathological abnormal findings were leiomyoma with cellular atypia (one case in the LM + TOHA group, two cases in the LM group) and one leiomyosarcoma in the LM group.

**Figure 4:**
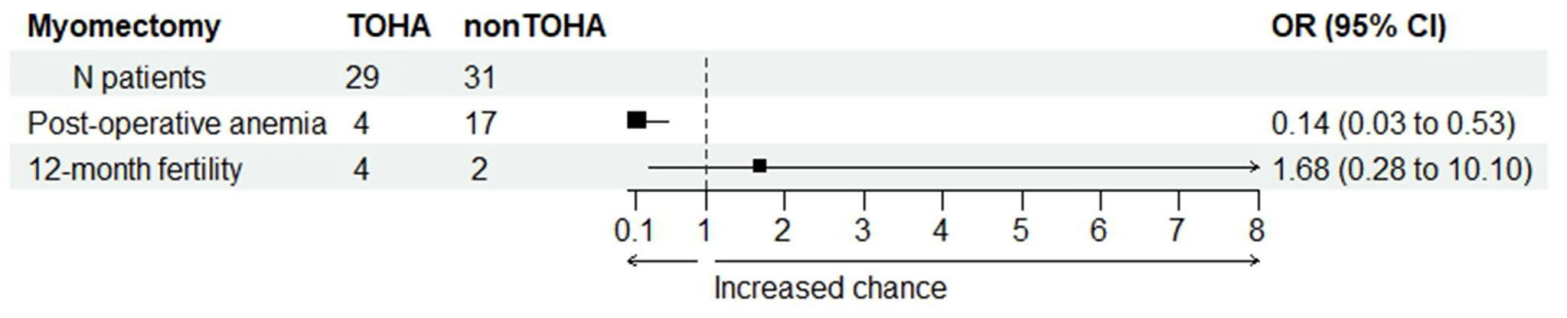
Comparison between the two study groups with regard to post-operative anemia and post-intervention fertility. Odds ratio (OR) values provide quantitative evidence towards favoring transient occlusion of hypogastric artery (TOHA) concerning both surgical outcomes.

The change in Hb was statistically significantly reduced in the “LM + TOHA” group compared to the “LM” group: 1.68 ± 0.67 (0.39–3.99) vs. 2.63 ± 1.06 (0.83–4.92), respectively (*p* < 0.001). Postoperative iron perfusion was significantly higher in “LM” group (*p* < 0.001), postoperative blood transfusion was marginally higher in “LM” group (*p* = 0.053), moderate to severe postoperative anemia was significantly higher in “LM” group (*p* = 0.001), and for the 12-month post-intervention fertility there were no differences between the two groups (*p* = 0.682) (Table 3). Figure 4 shows the balance between these latter two secondary aspects (namely, post-operative anemia and fertility) in terms of odds ratios. There is quantitative evidence favoring TOHA with regard to these categorical outcomes of LM surgery: there is more than 7 times less risk of post-operative anemia in the TOHA group (namely, 1/0.14 = 7.14); although lacking the statistical significance (95% CI includes 1 and is very large, therefore imprecise), there is a better chance of 12-month fertility in the TOHA group. One patient from the LM + TOHA group required conversion to laparotomy due to major bleeding which was difficult to manage, and from the LM group a patient who had seven leiomyomas. Subsequent follow-ups showed that there were no postsurgical complications. Additionally, none of the patients had to be readmitted, and there were no reports of any deaths.

**Table 3:** Study outcomes in the two groups of laparoscopic myomectomy (LM): with and without transient occlusion of hypogastric artery (TOHA).

	LM + TOHA	LM	*p*-value
Variable	*N* = 29	*N* = 31	
Change in hemoglobin level (g/dL) ^(a)^	1.68 ± 0.67 (0.39–3.99)	2.63 ± 1.06 (0.83–4.92)	<0.001**
Need for post-operative iron perfusion ^(b)^	0	12 (38.7%)	<0.001**
Need for post-operative blood transfusion ^(d)^	0	5 (16.1%)	0.053
Moderate to severe post-operative anemia ^(d)^	4 (13.8%)	17 (68.7%)	0.001**
12-month post intervention fertility ^(d)^	4 (13.8%)	2 (6.5%)	0.682

Data presented as mean ± standard deviation (min-max) for numerical variables, or *n* (%) for categorical variable. ^(a)^ *t*-test for independent samples; ^(b)^ asymptotic Chi-square test; ^(d)^ Chi-square, Monte Carlo simulation (10,000 samples). Statistical significance: *p < 0.05; **p < 0.01. LM, laparoscopic myomectomy; TOHA, transient occlusion of the hypogastric artery.

## 4. Discussion

Over the past few decades, there have been significant changes in the treatment of fibroids. Minimally invasive surgery offers several advantages over laparotomy, including quicker recovery, less postoperative pain, decreased morbidity and a more satisfactory cosmetic outcome (6). Predictors for postoperative pain after laparoscopic procedures and adequate management methods are an important topic in continuous development (17). However, the choice of surgical approach depends on factors such as the size and number of fibroids, surgeon’s skill and patient’s desire for fertility preservation.

To date, making an accurate preoperative differential diagnosis between benign uterine tumors and uterine malignancy has proven to be challenging, yet critical for the appropriate surgical management. Proper patient selection is key in minimizing the incidence of misdiagnosed sarcomas. Studies have demonstrated that adenomyosis can coexist with, and may even be the cause of, endometrial cancer (18). Since the preoperative diagnosis of malignant tumors remains a challenge, focus has shifted towards investigations that can distinguish cancer from adenomyosis, such as ultrasound evaluations of subendometrial vascularity (19). In our study, all patients underwent preoperative evaluations using ultrasound and other imaging methods as available. As an additional precaution, leiomyomas were excised using in-bag morcellation, with a tissue retention system for the fragments. This approach was used to restrict intraperitoneal tissue spread, in case the postsurgical histopathological examination indicated malignancy.

Despite the benefits offered by laparoscopy, maintaining hemostasis throughout the procedure can occasionally be challenging. Fortunately, several treatments are available for managing hemostasis during myomectomy, including uterotonics, vasopressin, antifibrinolytic drugs, gelatin-thrombin as a hemostatic sealant, peri-cervical mechanical tourniquet, or laser dissection (12, 13, 20, 21).

Around the turn of the century, Liu et al. (22) introduced a new technique of uterine artery occlusion (UAO), namely laparoscopic bipolar coagulation of uterine vessels. It was initially investigated as a standalone treatment option for leiomyomas in response to the potential complications given by uterine artery embolization (23, 24). Over the following decades, various approaches to UAO have been researched, including combinations between myomectomy and UAO with either permanent or transitory uterine vessel occlusion. This was achieved through coagulation, ligation using suture, or vessel clips (24–26). Preventive UAO during LM has been shown to reduce significantly intraoperative bleeding, minimize the risk of complications, and shorten hospital stay compared to LM alone (27–30). These results have been thoroughly confirmed in multiple systematic reviews over the past decade (31, 32). A more recent systematic review and meta-analysis published by Sanders in 2019 found compelling evidence that combining myomectomy with UAO has the benefit of significantly reducing surgery-associated blood loss without added complications (24).

In 2022, Hiratsuka et al. published a case–control study demonstrating that a temporary uterine artery ligation using sutures has the advantage of reducing intraoperative blood loss, while being less invasive than clipping (25). However, compared to other surgical approaches where clips were used, suturing was found to prolong the surgery time by an additional 40 min (25). One crucial surgical outcome of our study is that TOHA did not prolong the overall operating time, a finding which is consistent with other studies (20, 33).

Regarding concerns raised by some authors (25, 34) that uterine artery clipping might pose potential risks of masked bleeding after the removal of vascular clamps and the chance of promoting postoperative cervical hematoma, our study did not report any clipping-related injuries.

Even though hypogastric/internal iliac artery ligation has been recognized as a method for managing hemorrhage in obstetrics and gynecology since 1893, it was Peter Siegel who underscored its significance due to its remarkable potential to save lives (35). A form of temporary hypogastric artery occlusion using an endovascular balloon has been widely explored in combination with myomectomy or hysterectomy (36–39). Nonetheless, there is a gap in the literature concerning the use of vascular clips for temporary hypogastric artery occlusion during laparoscopic myomectomy. As far as the authors are aware, no other articles have discussed the application of this specific technique as depicted in this manuscript. Similar articles on this topic focus on the transitory occlusion of the uterine artery. Our analysis suggests that the anterior trunk of the hypogastric artery is more accessible for dissection and provides a safer clip application. Compared to the uterine artery, the anterior trunk of the hypogastric artery has a larger diameter and a thicker wall, which minimizes the risk of avulsion during clip removal.

TOHA’s effectiveness in reducing surgery-associated blood loss can be evaluated using parameters such as the postoperative drop in hemoglobin, intraoperative blood loss, and postoperative rate of transfusions. Measuring preoperative and postoperative hemoglobin levels provide an assessment of whether performing TOHA was effective in reducing blood loss. While intraoperative blood loss may be challenging to measure accurately due to the variability in irrigation fluid usage, postoperative Hb drop may constitute a more reliable indicator of the effectiveness of TOHA in reducing bleeding during surgery (34).

In our study, the primary outcome was surgery-associated blood loss as measured by the change in Hb level. We found that the laparoscopic TOHA at the time of LM, compared with LM alone, resulted in a statistically significant reduction in blood loss expressed as the mean differences in Hb measured before and after the surgery. The difference in Hb level (delta Hb) was significantly lower in the TOHA group, compared to the non-clipping approach: 1.68 ± 0.67 vs. 2.63 ± 1.06 (*p* < 0.001). Controlling operative bleeding is critical during surgical procedures, particularly those involving multiple or large fibroids, to avoid complications such as massive intraoperative bleeding and the need for conversion to laparotomy.

The rate of required iron perfusion or transfusions, other indirect measures of blood loss, can also be used to evaluate the effectiveness of TOHA. In our study, there was a statistically significant higher rate of postoperative iron perfusion in the non-clipping group (0 vs. 12 patients), with no statistical differences in the number of patients who required a postoperative blood transfusion.

The aim of this study was to evaluate the feasibility and effectiveness of our new and particular approach, TOHA, during laparoscopic myomectomy, in reducing surgery-associated blood loss. The novelty of our approach is represented by the placement of titanium clips at the level of the anterior trunk of the hypogastric artery, cranially to the emergence of the uterine artery. Benefits of this approach include easier and faster dissection, no need to open the broad ligament, and the ability to identify vascular structures under the parietal peritoneum on the pelvic side wall. Although TOHA adds another layer to the surgical technique during LM, our study showed it does not prolong operative times when compared to a standard LM.

This article describes an efficient method to minimize blood loss during conservative uterine surgery. Other types of surgical uterine-sparing procedures such as caesarean scar ectopic pregnancy, uterine arterio-venous fistulas or interstitial cornual pregnancies can also benefit from TOHA for minimizing intraoperative bleeding (40). As previously stated, TOHA has virtually the same applications as the laparoscopic temporary clipping of the uterine arteries, with some supplementary potential benefits (34).

The limitations of our investigation can be attributed to the single-center design, the fact that all procedures were performed by a single team, and due to hospital’s protocol, the estimation of blood loss was done by only measuring differences in hemoglobin. The strengths of our study come from the prospective design, being a proof of concept with the very advantage of consistency throughout all the surgical approach except for the technique used for the temporary artery occlusion, TOHA. The key strength is that we introduced this new technique for limiting blood loss with numerous potential applications for uterine-sparing procedures.

In conclusion, performing TOHA prior to LM provides numerous benefits, including reducing surgery-associated blood loss, minimizing the risk of complications, and lowering the occurrence of postoperative anemia. The technique does not significantly impact the operative time, making it a viable option for improving patient outcomes. Further studies are necessary to evaluate the impact of TOHA on fertility.

## Data availability statement

The raw data supporting the conclusions of this article will be made available by the authors, without undue reservation.

## Ethics statement

The studies involving human participants were reviewed and approved by Human Ethical Committee of the University of Medicine and Pharmacy “Victor Babes,” Timişoara, Romania. The patients/participants provided their written informed consent to participate in this study.

## Author contributions

LB, MP, SB, CS, DG, RC, and LP: conceptualization. LB, AP, MP, and LP: methodology. LB, CS, and LP: validation. AP, SN, and DL: formal analysis and visualization. CS and LB: investigation and resources. SN, AP, and DL: data curation. LB, AP, SN, DL, and LP: writing—original draft preparation. LB, MP, SN, AP, and DL: writing—review and editing. DL and LP: supervision. LB: funding acquisition. All authors contributed to the article and approved the submitted version.

## Funding

This research was funded by a doctoral research school grant (IMLHCV) from the Victor Babeş University of Medicine and Pharmacy Timişoara, number 44/10.12.2018, grant MLHCV.

## Conflict of interest

The authors declare that the research was conducted in the absence of any commercial or financial relationships that could be construed as a potential conflict of interest.

## Publisher’s note

All claims expressed in this article are solely those of the authors and do not necessarily represent those of their affiliated organizations, or those of the publisher, the editors and the reviewers. Any product that may be evaluated in this article, or claim that may be made by its manufacturer, is not guaranteed or endorsed by the publisher.
